# Effect of changing the acquisition trajectory of the 3D C-arm (CBCT) on image quality in spine surgery: experimental study using an artificial bone model

**DOI:** 10.1186/s13018-023-04394-0

**Published:** 2023-12-04

**Authors:** Maxim Privalov, Benno Bullert, Jula Gierse, Eric Mandelka, Sven Y. Vetter, Jochen Franke, Paul A. Grützner, Benedict Swartman

**Affiliations:** BG Klinik Ludwigshafen, Ludwig-Guttmann-Straße 13, 67071 Ludwigshafen am Rhein, Germany

## Abstract

**Background:**

Intraoperative 3D imaging using cone-beam CT (CBCT) provides improved assessment of implant position and reduction in spine surgery, is used for navigated surgical techniques, and therefore leads to improved quality of care. However, in some cases the image quality is not sufficient to correctly assess pedicle screw position and reduction, especially due to metal artifacts. The aim of this study was to investigate whether changing the acquisition trajectory of the CBCT in relation to the pedicle screw position during dorsal instrumentation of the spine can reduce metal artifacts and consequently improve image quality as well as clinical assessability on the artificial bone model.

**Methods:**

An artificial bone model was instrumented with pedicle screws in the thoracic and lumbar spine region (Th10 to L5). Then, the acquisition trajectory of the CBCT (Cios Spin, Siemens, Germany) to the pedicle screws was systematically changed in 5° steps in angulation (− 30° to + 30°) and swivel (− 30° to + 30°). Subsequently, radiological evaluation was performed by three blinded, qualified raters on image quality using 9 questions (including anatomical structures, implant position, appearance of artifacts) with a score (1–5 points). For statistical evaluation, the image quality of the different acquisition trajectories was compared to the standard acquisition trajectory and checked for significant differences.

**Results:**

The angulated acquisition trajectory increased the score for subjective image quality (*p *< 0.001) as well as the clinical assessability of pedicle screw position (*p *< 0.001) highly significant with particularly strong effects on subjective image quality in the vertebral pedicle region (*d *= 1.06). Swivel of the acquisition trajectory significantly improved all queried domains of subjective image quality (*p *< 0.001) as well as clinical assessability of pedicle screw position (*p *< 0.001). The data show that maximizing the angulation or swivel angle toward 30° provides the best tested subjective image quality.

**Summary:**

Angulation and swivel of the acquisition trajectory result in a clinically relevant improvement in image quality in intraoperative 3D imaging (CBCT) during dorsal instrumentation of the spine.

## Introduction

Dorsal instrumentation with pedicle screws can be considered as gold standard in surgical treatment of traumatic and degenerative spinal fractures [1, 2]. It can be performed by open or percutaneous/minimal-invasive approach [3]. Computer-navigated percutaneous surgical techniques are also increasingly used [4] to improve the quality of care.

Although pedicle screw surgery is considered very safe, and the rate of clinically relevant complications is low [5], the topographic proximity to the spinal cord, nerves and blood vessels means that there is always the potential for accidental damage to these structures from malpositioned pedicle screws [5, 6].

In addition to postoperative radiographic control, postoperative CT has been established for verification of reduction and implant position of pedicle screws [7].

Intraoperatively, mobile 2D C-arms are usually used for imaging. However, assessment of reduction and pedicle screw location using 2D fluoroscopy is limited in some cases [8]. Modern C-arms have the capability of 3D imaging using CBCT, which allows better intraoperative control of pedicle screw position [9]. Possible injuries of the vertebral pedicle cortical can be detected just as well as by postoperative CT [10].

A recent study reports immediate intraoperative revision due to screw malposition in 7% of pedicle screws, based on intraoperative CBCT imaging [11]. In the treatment of trauma-related spinal injuries, pedicle screw malposition in the lumbar spine was significantly reduced by intraoperative CBCT compared to 2D fluoroscopy [12]. Furthermore, intraoperative 3D imaging can help to reduce the number of revision procedures and shorten surgery time [13].

However, due to metal artifacts, which inevitably occur due to the inserted pedicle screws, the assessability of the CBCT images may be limited. In individual cases, this may result in insufficient intraoperative image quality to reliably assess pedicle screw position [14].

Currently so-called MAR (metal artifact reduction) algorithms are used to improve image quality when artifacts in CBCT occur due to metal implants [15, 16].

The purpose of this study is to evaluate whether a change of the acquisition trajectory of the CBCT in relation to the pedicle screws leads to a reduction of metal artifacts and therefore to improvement of image quality and clinical assessability in spine surgery?

Hence the hypothesis of this study is: By changing the acquisition trajectory of the CBCT during spine surgery, metal artifacts will be reduced and consequently image quality as well as clinical assessability will be improved.

## Methods

A total of 16 pedicle screws (8 pedicle screw pairs) were inserted into an artificial bone model of the spine with soft tissue sheath (Synbone, Zizers, Switzerland) in the vertebral bodies Th10 to L5. For this purpose, a CT of the artificial bone model was acquired using the Airo system (Brainlab AG, Munich, Germany) and subsequently instrumented via the dorsal approach using curve navigation (Brainlab AG, Munich, Germany) by an experienced spine surgeon. 3D datasets were created of each vertebra at 13 pedicle screw angulation angles and 13 pedicle screw swivel angles (except for L5: here + 30° pedicle screw angulation could not be achieved due to collision of artificial bone and 3D C-arm).

The mobile 3D C-arm “Cios Spin” (Siemens, Erlangen, Germany) was used to acquire the datasets.

### Definition of terms

Standard acquisition trajectory: An implant angle of 0° pedicle screw angulation and 0° pedicle screw swivel is used as the standard acquisition trajectory. To achieve this acquisition trajectory, the pedicle screw pair to be examined was positioned accordingly in the beam path, resulting in different initial positions of the artificial bone model for each vertebral body.

Orbital position: In this study orbital position refers to the position of the C-arm in orbital rotation. The 0° orbital position corresponds to an anterior–posterior (AP) image, the 90° orbital position to a lateral image.

**Fig. 1:**
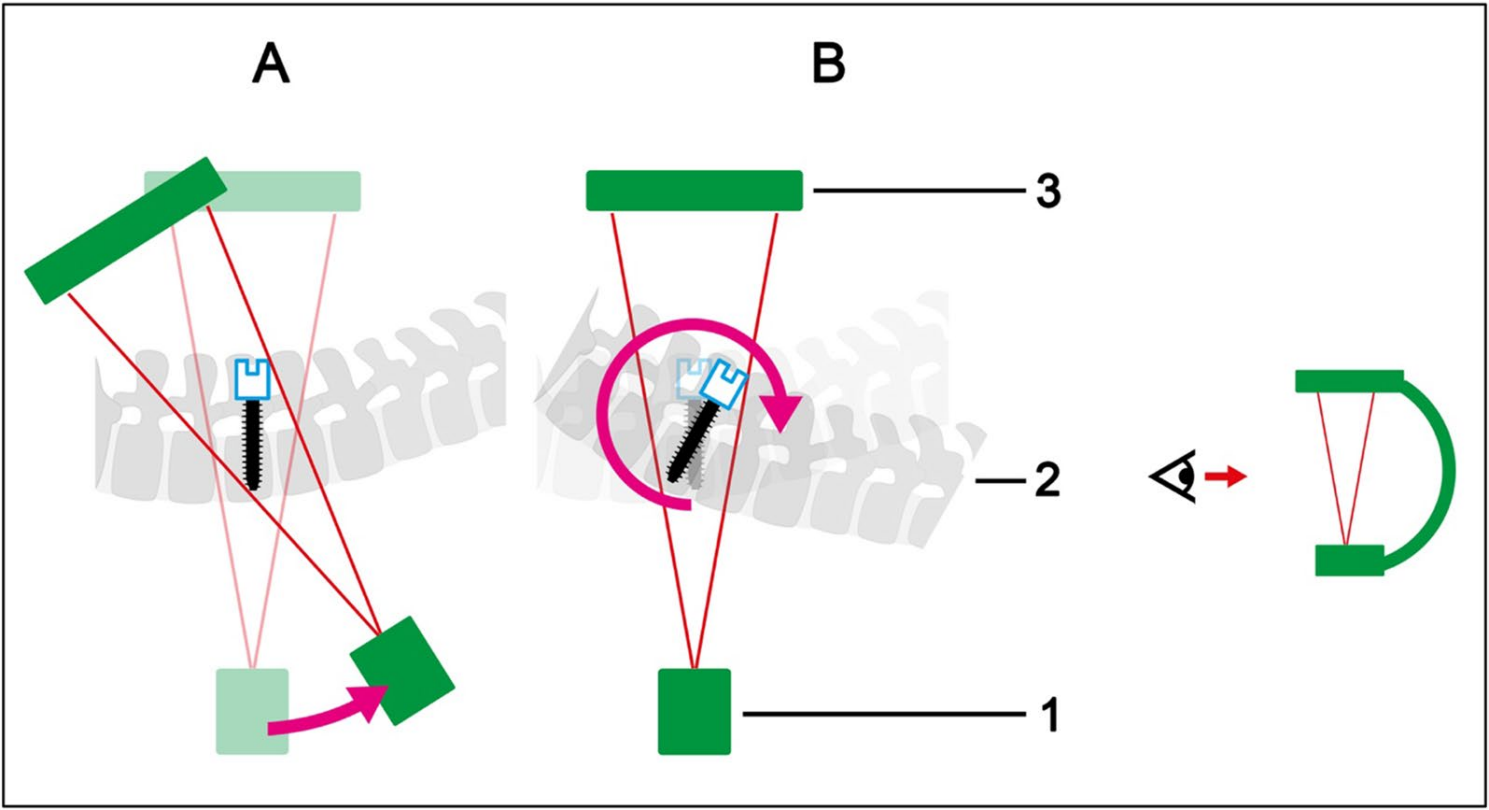
Angulation of C-arm (**A**) and pedicle screws (**B**). 1: Radiation source C-arm, 2: spine with pedicle screws, 3: detector C-arm

Angulation (Fig. 1): Angulation refers to the rotation of the pedicle screws in the sagittal plane and can be achieved both by angulating the 3D C-arm in relation to the examining pedicle screws (hereinafter: C-arm angulation) and by angulating the pedicle screws in relation to the 3D C-arm (hereinafter: pedicle screw angulation).

**Fig. 2:**
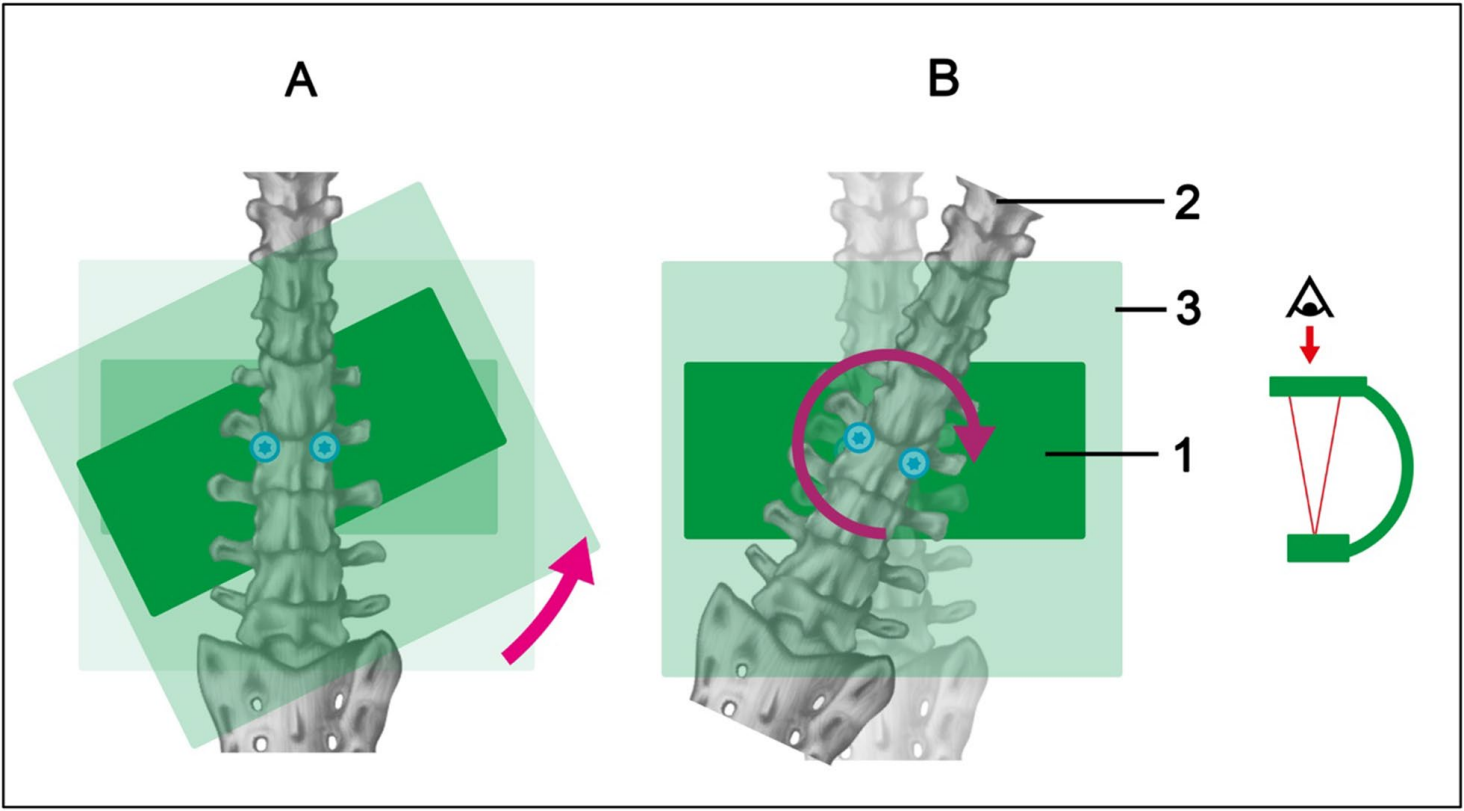
Swivel of C-arm (**A**) and pedicle screws (**B**). 1: Radiation source C-arm, 2: spine with pedicle screws, 3: detector C-arm

Swivel (Fig. 2): Swivel is the rotation of the pedicle screws in the frontal plane. The swivel of the pedicle screws in the beam path can be achieved both by swiveling the 3D C-arm in relation to the examining pedicle screws (hereinafter: C-arm swivel) and by swiveling the pedicle screws in relation to the 3D C-arm (hereinafter: pedicle screw swivel)

### Setup angulation

**Fig. 3:**
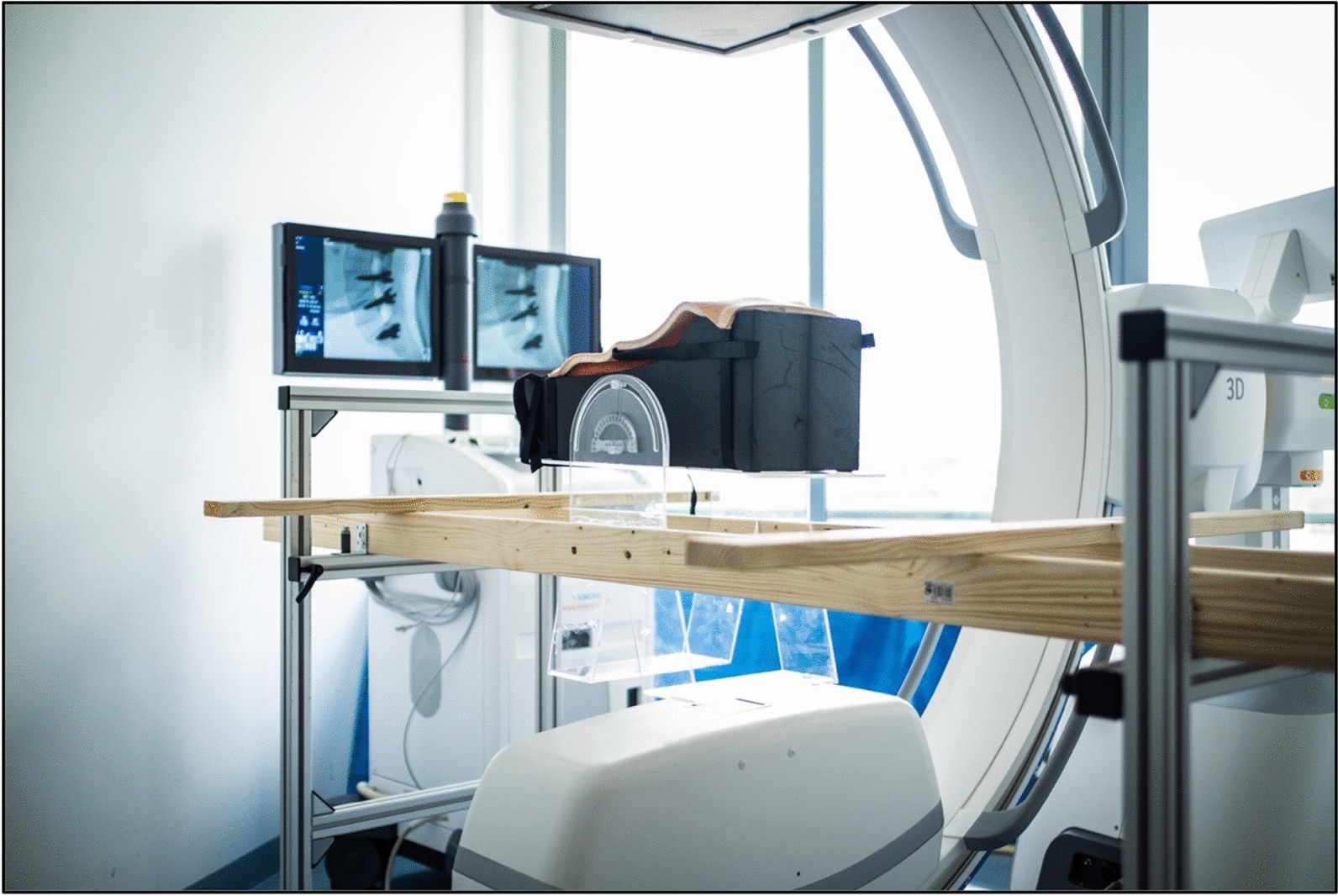
Experimental setup of pedicle screw angulation

The tilt table was made of polymethyl methacrylate (acrylic glass), as this material does produce almost no additional artifacts in the ROI (region of interest). The tilt table was attached to a wooden substructure. The artificial bone model was fixed in the corresponding foam tray on the tilt table using Velcro straps (Fig. 3).

The C-arm angulation of the “Cios spin” is limited to 15° for the acquisition of 3D scans. By using additional calibrations, extending the angulation range of the 3D C-arm is potentially possible. To extend the angulation range and to eliminate differences in image quality due to calibration the angulation was adjusted using only the tilt table.

Subsequently, pedicle screw angulation was adjusted in 5° increments from − 30° to + 30° in relation to the standard acquisition trajectory. In each position, a lateral image (90° orbital position) and an AP image (0° orbital position) were taken to check the position of the pedicle screw pair for correct location in the iso-center of the 3D C-arm.

### Setup swivel

The acrylic tilt table from the angulation experiments was now converted to a rotary table to adjust the artificial bone model in pedicle screw swivel. For this purpose, a base plate was fixed on an examination table with a carbon fiber work plate. After that the rotation plate was attached to this base plate via a screw, which served as a pivot. This rotation plate carried the artificial bone model with foam shell.

Subsequently the pedicle screw swivel was adjusted in 5° increments from − 30° to + 30° in relation to the standard acquisition trajectory. In each position, a lateral image (90° orbital position) and an AP image (0° orbital position) were taken to check the position of the pedicle screw pair for correct location in the iso-center of the 3D C-arm.

### Radiological analysis

First, the 3D datasets were sorted by angle in the OsiriX Lite Viewer (Pixmeo SARL Company, Switzerland). Then, the metadata were removed from the 3D datasets and replaced by a blinded 5-digit code, which allowed a unique assignment of the 3D datasets in retrospect.

Hereupon, in a randomized manner by 3 independent examiners, the radiological evaluation regarding the image quality was performed by means of a questionnaire.

The questionnaire consists of 9 statements to be evaluated (hereafter referred to as "questions"), which cover different parts of the image quality:

1. The dorsal vertebral body surface can be fully assessed.
2. Fragments in the spinal canal can be definitively excluded.
3. The width of the spinal column can be clearly determined.
4. The edges of the vertebral pedicles can be fully assessed.
5. Screw perforations of the vertebral pedicles can be clearly assessed.
6. The width of the screws can be reliably determined in relation to the width of the pedicles.
7. The length of the screws can be clearly determined.
8. Ventral perforation of the screws can be reliably assessed.
9. The image quality allows a reliable assessment from a clinical point of view.

These questions can be divided into four categories:

- *Category 1* Questions 1–3 consider image quality of the dorsal vertebral body and spinal canal.
- *Category 2* Questions 4–6 consider the image quality of the vertebral pedicles.
- *Category 3* Questions 7–8 consider screw length and the image quality of the ventral vertebral body.
- *Category 4* Question 9 asked about clinical assessability.

The mean score over the entire questionnaire (Q1–Q9) is used as a measure of “subjective image quality.” Individual questions were assessed with a score from 1 to 5, with a score of 1 representing the worst score and a score of 5 representing the best score. Radiological subjective assessment of metal artifacts on CBCT has been performed several times in the past using a 5-point scale [17, 18]. The investigators were a senior physician (deputy section chief of acute traumatology) with a residency in trauma surgery and orthopedics and an additional designation in special trauma surgery, a resident in trauma surgery and orthopedics in his 6th year of training, and a 5th-year medical student. Both physicians have extensive experience in intraoperative 3D imaging, both in spine surgery and in other areas of trauma surgery.

### Statistical analysis

The program SPSS version 26 (IBM Company, Armonk, USA) was used for statistical analysis. In the test series "pedicle screw angulation," the effect of the trajectory on the subjective image quality was determined using a repeated-measures analysis of variance (ANOVA). In the test series "pedicle screw swivel" the preexisting angulation of the pedicle screws was also included as a covariate in the ANOVA.

A *p* value < 0.05 is assumed to be statistically significant (corresponding to 95% significance level). A *p* value < 0.01 is considered highly significant.

Cohen's d is used as a measure of effect size. The value is interpreted as follows:

-  < 0.5—small effect; 0.5–0.8—medium effect; > 0.8—large effect.

For analysis of the intraclass correlation coefficient (ICC) the software R (R Foundation for Statistical Computing, Vienna, Austria) was used. The value is interpreted as recommended by Cicchetti [19]: < 0.40—poor; 0.40–0.59—fair; 0.60–0.74—good; 0.75–1.00—excellent.

## Results

### Descriptive data

3D datasets were generated from 8 pedicle screw pairs in 13 pedicle screw angulation angles and 13 pedicle screw swivel angles (except for L5 30° pedicle screw angulation due to collision of artificial bone and 3D C-arm). Thus, 103 3D datasets were created in the pedicle screw angulation study and 104 3D datasets were created in the pedicle screw swivel study.

### Standard acquisition trajectory

In the standard acquisition trajectory the average subjective image quality (corresponding to the mean score over all questions) was 2.83 ± 1.11. Due to the wide variation between the scores of the cranial and caudal spinal levels, two spine areas (Th10–L1 and L2–L5) were defined in the analysis of the datasets. Image quality in the standard acquisition trajectory of spine area 1 (Th10–L1) averaged 2.04 ± 0.89, while spine area 2 (L2–L5) achieved a mean of 3.62 ± 0.65.

### Angulation

The direction of pedicle screw angulation has no significant effect on subjective image quality (*p *= 0.728); therefore, absolute values of the angular change are used for evaluation.

**Fig. 4:**
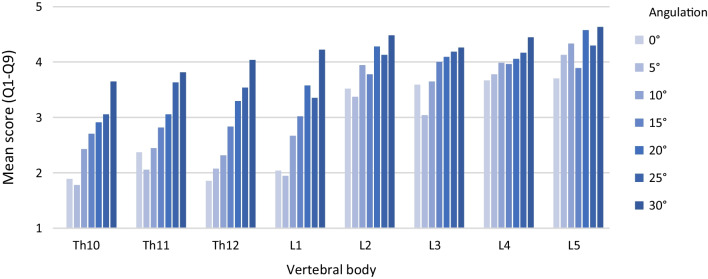
Subjective image quality (mean score (Q1–Q9)) in relation to pedicle screw angulation

Subjective image quality can be significantly improved by angulation of the acquisition trajectory to the pedicle screws with increasing angle (*M*_0°_ = 2.83 ± 1.11; *M*_30°_ = 4.19 ± 0.54; *p *< 0.001). The best subjective image quality was always achieved at an angulation of 30°. As can be seen in Fig. 4, there is a variation between the different spine regions due to the effect of the pedicle screw angulation. A detailed list of all significances and effect sizes for the angulation experiments can be found in Table 1.

**Table 1:** Effect of pedicle screw angulation versus standard acquisition trajectory

	*p*	${\eta }_{p}^{2}$	Cohens *d*
*Overall (Th10*–*L5)*			
Subjective image quality (Q1–Q9)	< 0.001	0.481	0.96
Spinal canal and dorsal vertebral body (Q1–Q3)	0.017	0.131	0.39
Vertebral pedicle (Q4–Q6)	< 0.001	0.531	1.06
Screw length and ventral vertebral body (Q7–Q8)	< 0.001	0.522	1.05
Clinical assessability (Q9)	< 0.001	0.443	0.89
*Spine area 1 (Th10*–*L1)*			
Subjective image quality (Q1–Q9)	< 0.001	0.624	1.29
Spinal canal and dorsal vertebral body (Q1–Q3)	0.029	0.239	0.56
Vertebral pedicle (Q4–Q6)	< 0.001	0.697	1.52
Screw length and ventral vertebral body (Q7–Q8)	0.001	0.597	1.22
Clinical assessability (Q9)	< 0.001	0.601	1.23
*Spine area 2 (L2*–*L5)*			
Subjective image quality (Q1–Q9)	0.003	0.368	0.76
Spinal canal and dorsal vertebral body (Q1–Q3)	0.209	0.117	
Vertebral pedicle (Q4–Q6)	0.003	0.368	0.76
Screw length and ventral vertebral body (Q7–Q8)	0.003	0.475	0.95
Clinical assessability (Q9)	0.007	0.323	0.69

**Fig. 5:**
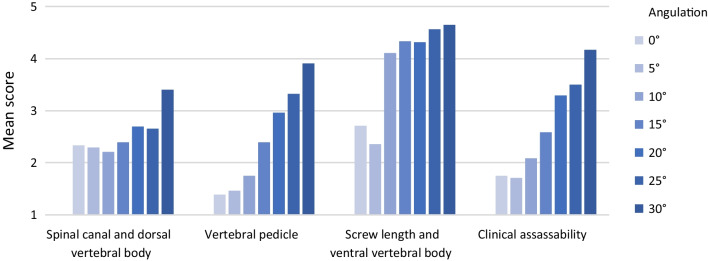
Categories in spine area 1 (Th10–L1)—Angulation

Over the vertebral bodies Th10—L1 (spine area 1), there is a significant increase in subjective image quality due to pedicle screw angulation (*p *< 0.001). In all four categories the score is significantly improved by pedicle screw angulation (Fig. 5): vertebral pedicle (*p *< 0.001); screw length and ventral vertebral body (*p *= 0.001), clinical assessability (*p *< 0.001), spinal canal and dorsal vertebral body (*p *= 0.029).

**Fig. 6:**
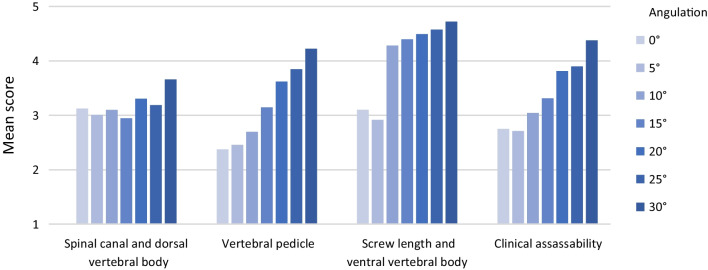
Categories in spine area 2 (L2–L5)—Aangulation

Over the vertebral bodies L2–L5 (spine area 2), there is also a significant effect of pedicle screw angulation on subjective image quality (*p *= 0.003). In 3/4 categories a significant increase of the score by pedicle screw angulation can be observed (Fig. 6): Vertebral pedicle (*p *= 0.003); screw length and ventral vertebral body (*p *= 0.003); clinical assessability (*p *= 0.007); spinal canal and dorsal vertebral body (*p *= 0.209).

### Swivel

Due to the anatomy of the spine, the pedicle screws in the images of the "pedicle screw swivel" experiment also have a pedicle screw angulation angle (Table 2), which was predetermined by the position of the pedicles and could not be compensated by the rotation table. The following table shows the pedicle screw angulation in the respective vertebral body.

**Table 2:** Angulation in prone position

Vertebral body	Th10	Th11	Th12	L1	L2	L3	L4	L5
Angulation	1°	0°	9°	1°	17°	12°	20°	48°

The direction of pedicle screw swivel has no significant effect on subjective image quality (*p *= 0.922); therefore, absolute values of the angular change are used for evaluation.

**Fig. 7:**
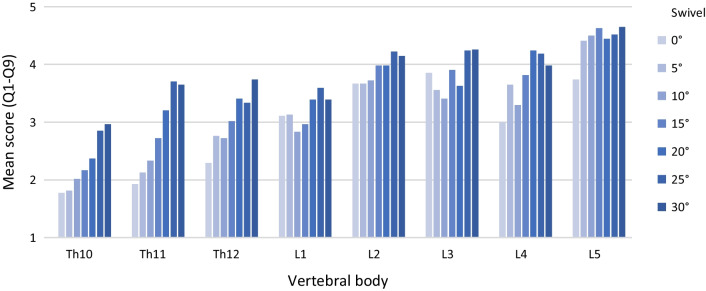
Subjective image quality (mean score (Q1-Q9)) in relation to pedicle screw swivel

Subjective image quality can be significantly improved with increasing angle by pedicle screw swivel (*M*_0°_ = 2.92 ± 1.05; *M*_30°_ = 3.85 ± 0.78; *p *< 0.001). As can be seen in Fig. 7, the influence of pedicle screw swivel on subjective image quality also shows variation between different areas of the spine. A detailed list of all significances and effect sizes for the swivel experiments can be found in Table 3.

**Table 3:** Effect of pedicle screw swivel versus standard acquisition trajectory

	*p*	${\eta }_{p}^{2}$	Cohens *d*
*Overall (Th10–L5)*			
Subjective image quality (Q1–Q9)	< 0.001	0.483	0.97
Spinal canal and dorsal vertebral body (Q1–Q3)	< 0.001	0.347	0.73
Vertebral pedicle (Q4–Q6)	< 0.001	0.402	0.82
Screw length and ventral vertebral body (Q7–Q8)	< 0.001	0.301	0.66
Clinical assessability (Q9)	< 0.001	0.341	0.72
*Spine area 1 (Th10–L1)*			
Subjective image quality (Q1–Q9)	< 0.001	0.652	1.37
Spinal canal and dorsal vertebral body (Q1–Q3)	< 0.001	0.554	1.11
Vertebral pedicle (Q4–Q6)	< 0.001	0.498	1.00
Screw length and ventral vertebral body (Q7–Q8)	0.001	0.433	0.87
Clinical assessability (Q9)	< 0.001	0.542	1.09
*Spine area 2 (L2–L5)*			
Subjective image quality (Q1*–*Q9)	0.010	0.239	0.56
Spinal canal and dorsal vertebral body (Q1*–*Q3)	0.119	0.180	
Vertebral pedicle (Q4*–*Q6)	0.025	0.208	0.51
Screw length and ventral vertebral body (Q7*–*Q8)	0.288	0.112	
Clinical assessability (Q9)	0.109	0.155	

**Fig. 8:**
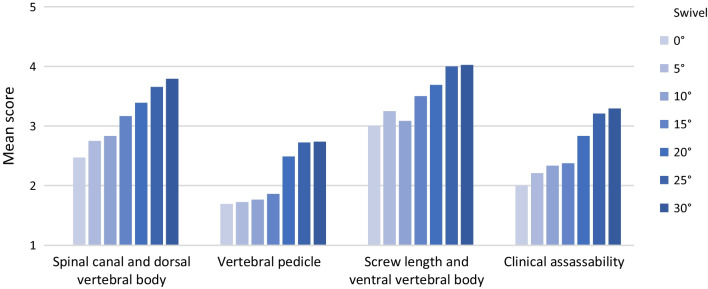
Categories in spine area 1 (Th10–L1)﻿—Swivel

Over the vertebral bodies Th10–L1 (spine area 1) a significant improvement of the subjective image quality by pedicle screw swivel is shown (*p *< 0.001). Pedicle screw swivel also has a significant positive effect on all categories of the questionnaire (Fig. 8): vertebral pedicle (*p *= 0.000); screw length and ventral vertebral body (*p *= 0.001), clinical assessability (*p *< 0.001); spinal canal and dorsal vertebral body (*p *< 0.001).

**Fig. 9:**
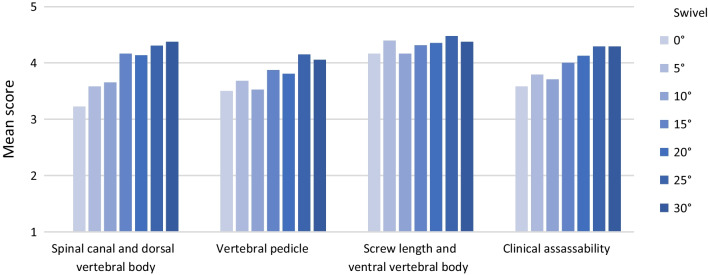
Categories in spine area 2 (L2–L5)—Swivel

Over the vertebral bodies L2–L5 (spine area 2), there is also a significant effect of pedicle screw swivel on subjective image quality (*p *= 0.010). While the category "vertebral pedicle" is significantly improved by pedicle screw swivel (*p *= 0.025), no significant increase of the score is observed in the remaining categories: screw length and ventral vertebral body (*p *= 0.228); clinical assessability (*p *= 0.109); spinal canal and the dorsal vertebral body (*p *= 0.119) (Fig. 9).

### ICC

The intraclass correlation coefficient between the 3 raters is 0.537 for swivel and 0.496 for angulation (Table 4). Thus, it can be judged as "fair" in each case.

**Table 4:** ICC of swivel and angulation test series

	Swivel	Angulation
ICC	0.537	0.496
*p*	< 0.001	< 0.001
95% CI	0.427 < ICC < 0.64	0.381 < ICC < 0.605

## Discussion

The purpose of this study was to determine whether changing the acquisition trajectory of the CBCT in relation to the pedicle screw position could reduce metal artifacts and consequently improve image quality and clinical assessability of the screw position on the artificial bone model.

**Fig. 10:**
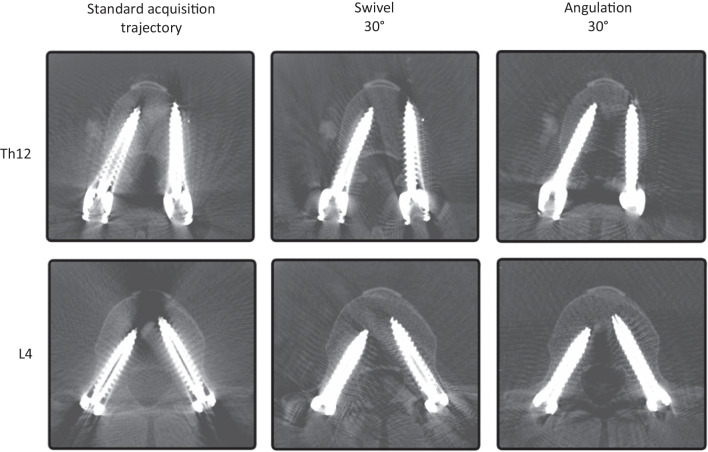
Visual examples of pedicle screws in Th12 and L4

In summary, it was shown that maximizing the angular change of the acquisition trajectory, both by swivel and angulation, can highly significant increase subjective image quality and clinical assessability (visual examples in Fig. 10).

### Pedicle screw angulation

Over the entire spinal area examined, image quality is highly significant improved by increasing pedicle screw angulation in all anatomical regions evaluated. 

The worst subjective image quality is achieved with a pedicle screw angulation of 5° in Th10 (1.78 ± 0.76). The best subjective image quality was achieved by L5 with a pedicle screw angulation of 30° (4.63 ± 0.55).

The effect of changed acquisition trajectory is strongest on the vertebral pedicles (*d *= 1.06), especially in the thoracolumbar transition (spine area 1) (*d *= 1.52).

A view at the vertebrae examined shows: Looking at the delta between the pedicle screw angulation angle with the worst and best subjective image quality score, L1 benefits the most (*Δ *= 2.28) and L4 the least (*Δ *= 0.77) from the changed acquisition trajectory.

Angulated acquisition trajectories, often referred to as scan planes, have already been described in the literature for CT. They are used to spare radiosensitive structures with a tilted scan plane [20] or to minimize hardening artifacts caused by bone in skull imaging [21].

There are also studies on the influence of angulated scan planes in CBCT. Zhao et al. [22] found that in cervical spine imaging, angulation of the scan axis by − 35° nearly halved the incidence of image noise and doubled the contrast-to-noise ratio in subsets. This examination also focuses on avoiding radiopaque bony structures.

Related to the avoidance of metal artifacts in CBCT, Wu et al. [23] were able to develop an algorithm that can reduce the occurrence of "blooming" artifacts by 46–70%. Again, angulation of the acquisition trajectory is a component contributing to this positive result. However, since this angulation is subject to dynamic change via the orbital rotation of the 3D C-arm, no fixed angulation angle for optimal artifact reduction can be drawn from the study of Wu et al.

The study situation shows that angulation of the acquisition trajectory can have a positive impact on image quality as well as metal artifact reduction in both CT and CBCT. Minimizing the amount of metal in the beam path by altering the acquisition trajectory also reduces the likelihood of photoelectric effect [24] and beam hardening [25], leading to a reduction in artifacts.

Previous studies, reporting angulation angles, always referred to the change from the 0° setting on the device, but not to the angle with respect to the metal implant examined. Only in the study by Wu et al. the position of the metal implants was considered, but without outputting a defined angulation angle.

Exact comparisons between previous work on angulation in CBCT and this study are not possible because in this study the angulation angle was set in relation to the examining pedicle screws. Also, for the first time the focus was on an evaluation specific to every vertebral body and performed by qualified observers from a clinical point of view.

In several studies it has been shown that 3D imaging in spine surgery reduces the incidence of revision surgery. A recent analysis by Zimmermann et al. [11] reports that malposition was detected in 7% of inserted pedicle screws because of intraoperative CBCT, which led to intraoperative revision. The rate of revision surgery decreases significantly with navigated instrumentation using 3D-CBCT compared with the freehand technique using 2D fluoroscopy, from 4.38% to 1.35% [26].

It is likely that the use of angulated acquisition trajectories and the resulting improved image quality will reduce the rate of revision surgery even further than it has already been possible with the use of intraoperative 3D imaging versus 2D imaging.

### Pedicle screw swivel

The purpose of the test series with the artificial bone model was to investigate the influence of the pedicle screw swivel on the subjective image quality. Due to the design, there was no possibility of compensating for the anatomically induced angulation of the pedicle screws in prone position. Thus, in this series of experiments, the influence of pedicle screw angulation on image quality varied depending on the initial position.

Pedicle screw swivel highly significantly improves image quality in spine area 1 (Th10–L1) in all categories of the questionnaire. In spine area 2 (L2–L5), however, there is only a significant improvement in the category of “vertebral pedicles.” Considering the effect sizes it also becomes apparent that even the significant results in spine area 2 are very weak compared to spine area 1.

These different results are caused by the fact that in spine area 1 the influence of the pedicle screw angulation was still small, because they were relatively close to the 0° of the standard acquisition trajectory (1°, 0°, 9°, 1°) in spine region 2; on the other hand, significantly stronger pedicle screw angulation angles (17°, 12°, 20°, 48°) and correspondingly stronger effects on the subjective image quality were seen. 

The fact that the effect in spine region 2 is so small is also evident in the comparison of the subjective image quality between 0° pedicle screw swivel and 30° pedicle screw swivel. While in spine region 1 the subjective image quality in the baseline situation at 0° pedicle screw swivel always reached values in the lower range of the score (< 3) on average, in spine region 2 it was permanently above the score of 3 on average. Corresponding to the different baseline situation, the increase in quality over the increasing pedicle screw swivel angle turns out to be smaller. The effect size remains small.

It can be assumed that the strong influence of pedicle screw angulation, as already seen in the experiments on pedicle screw angulation on the artificial bone model, has ensured the good image quality at 0° pedicle screw swivel in spine region 2 and thus reduces the possibilities for increasing the subjective image quality by pedicle screw swivel.

To investigate this further, future studies would need to examine the controlled combination of pedicle screw angulation and pedicle screw swivel.

A literature review did not reveal any relevant results on the influence of swivel on the image quality of 3D datasets. This is also due to the fact that common, stationary CT scanners do not offer direct adjustment options to change the swivel.

In CBCT, intraoperatively, the 3D C-arm is probably already frequently run with a swivel angle over the pair of pedicle screws to be examined due to the limited space available. However, there are no measurements or experiments on the extent to which this angle influences image quality and the occurrence of metal artifacts.

A possible explanation for the improved image quality due to swivel could be that with precise positioning of the 3D C-arm, the central beam no longer runs centrally through both pedicle screws but radiates between them. As a result, there is less metal in the beam path and the image quality increases. This space between the two pedicle screws depends on the size of the respective vertebral body, which means that it is not always easy to target.

Compared to changing the angulation, changing the swivel of the 3D C-arm has the advantage that there is no need to intervene in the calibration of the 3D C-arm. In addition, the adjustment can be done faster and without significantly more effort than with normal intraoperative 3D imaging.

The advantage of the swivel is that there is no change at the C-arm settings compared to the normal CBCT workflow. The C-arm simply has to be moved "diagonally" over the ROI.

The disadvantage, however, is that the influence of the swivel on the reduction of metal artifacts is less effective than using the more complex angulation.

### Different image quality of the spinal regions

In the "pedicle screw angulation" test series, as in the "pedicle screw swivel" test series, there is a dichotomy of subjective image quality in the starting position (standard acquisition trajectory). Consequently, there is a different potential for increase in image quality, which is the reason for a separate evaluation of two spine areas (Th10–L1 and L2–L5) with regard to significance and effect strength.

In the "pedicle screw swivel" test series, the different increase potentials in the spinal regions can be explained by the influence of the present pedicle screw angulation as described above.

In the “pedicle screw angulation” test series, there is no obvious covariate between the two areas. At this point it is helpful to take a look at the datasets created. In spine area 2, the occurrence of metal artifacts was also subjectively significantly minimized by pedicle screw angulation. However, due to the increasing size of the vertebral pedicles in area 2 compared to area 1, the metal artifacts in area 2 only slightly mask the pedicle cortical bone, which is why the assessability of this clinically very relevant structure remains good even without angulation adjustment, even in the presence of severe metal artifacts.

### Clinical relevance

The remarkable influence of pedicle screw angulation as well as pedicle screw swivel on subjective image quality and consequently on the score for "assessability from a clinical point of view" shows that changes in acquisition trajectory are of clinical relevance.

Improved intraoperative image quality allows more precise detection of pedicle cortical injuries and better assessment of screw perforations. Especially in the thoracolumbar transition, which is characterized by relatively narrow vertebral pedicles compared to the lumbar spine, the increase in image quality can make a decisive contribution to the reliable assessment of screw position. The fact that most spinal injuries also occur in this region [3] further highlights the relevance.

The intraoperative use of CBCT can significantly lower the postoperative revision rate, reduce the radiation exposure for the OR staff, and minimize potential postoperative complications. At the same time, however, there are individual cases in which the intraoperative image quality of CBCT is insufficient to reliably assess the position of the screw due to artifacts [14]. This has been observed especially in combination with small vertebral pedicles, where the pedicle cortex has a small distance to the metallic implant.

Due to the significant increase in subjective image quality because of pedicle screw angulation and pedicle screw swivel, it can be assumed that the reported positive aspects of intraoperative CBCT can be further extended by optimizing image quality.

### Clinical implementation

Angulation as well as swivel of the acquisition trajectory leads to significantly improved image quality in intraoperative 3D imaging (CBCT) with pedicle screws in situ. Implementation of this knowledge into daily surgical practice requires little additional effort. Sufficient angulation can be achieved by tilting the 3D C-arm or the operating table and by combining with the anatomically determined pedicle screw angulation.

With the Swivel, even less effort is required, as the 3D C-arm can simply be moved at an angle over the pedicle screws.

### Limitations of pedicle screw angulation

For more precise adjustment of the pedicle screw angulation angles and to extend the range to 30°, a tilt table was used, which rotated the pedicle screws in relation to the 3D C-arm.

In the intraoperative setting, this is not possible to this extent with the aid of the operating table; here, the pedicle screw angulation may have to be adjusted or extended via the 3D C-arm. However, this C-arm angulation is currently only possible to an extent of 15° in the case of the Siemens "Cios Spin" device used.

Although the 15° 3D C-arm angulation initially appears to be a major limitation in relation to the possible quality improvement with 30° pedicle screw angulation, this problem appears less relevant on closer examination: On the one hand, many pedicle screws are already angulated in the beam path, which means that an addition of 15° should generally result in sufficient pedicle screw angulation in the beam path. Alternatively, the angle can be further increased by tilting the operating table additionally. On the other hand, C-arm angulation of up to 30° is also technically possible with the “Cios Spin” 3D C-arm. However, this is currently limited to 15° due to the lack of dynamic calibration.

### Limitations of pedicle screw swivel

Due to the experimental setting, the angulated position of the pedicle screws could not be compensated for in the pedicle screw swivel experiments with the artificial bone model. Consequently, pedicle screw angulation also contributes to the image quality, but this was considered in the statistical evaluation.

### Limitations of the artificial bone model

The results of experiments on the artificial bone model may differ from results obtained in a study on a human specimen or on a patient. Although the artificial bone model has a soft tissue sheath and was developed specifically for the medical imaging field, the transferability of results to patient use may be limited.

### Limitations of the raters

Rater agreement was in the "fair" range for both swivel and angulation. Several factors have an influence here. Due to the relatively narrow scale with 5 points per question, even small deviations are associated with a strong decrease in the ICC.

While the ICC is also frequently used for metric measurements and can reach correspondingly high values here, the present data collection is a subjective assessment of the data. Therefore, values cannot be expected to be as high as with a metric measurement.

## Conclusion

Angulation as well as swivel of the acquisition trajectory leads to improved image quality in intraoperative 3D imaging (CBCT) while keeping the iso-center constant, which is confirmed by the outstanding effect size, especially in the case of angulation in the thoracolumbar region.

For the assessment of single pedicle screw pairs located in the iso-center, this study can demonstrate that in cases of unclear screw location, difficult assessment due to thin vertebral pedicles, or poor image quality, a modified acquisition trajectory can provide the decisive contribution for an intraoperative revision decision.

With improved intraoperative imaging capabilities, potentially fewer revision surgery can be expected, leading to improved patient outcome.

## Data Availability

The data and material are available from the corresponding author on reasonable request.
